# *Steringophorus merretti* n. sp. (Digenea: Fellodistomidae) from the deep-sea fish *Cataetyx laticeps* Koefoed (Ophidiiformes: Bythitidae) from the Goban Spur, Northeastern Atlantic Ocean

**DOI:** 10.1007/s11230-020-09919-3

**Published:** 2020-06-03

**Authors:** Rodney A. Bray, Andrea Waeschenbach

**Affiliations:** Department of Life Sciences, Natural History Museum, Cromwell Road, London, SW7 5BD UK

## Abstract

A new species of deep-sea digenean, *Steringophorus merretti* n. sp., is described from the bythitid fish *Cataetyx laticeps* in deep waters of the Goban Spur, Northeastern Atlantic. It is distinguishable from other described members of the genus by its tiny eggs and large cirrus-sac. A phylogenetic tree, based on 28S rDNA sequences, indicates that this species is embedded within a clade of deep-sea species and is sister to the eurybathic species *S. thulini* Bray & Gibson, 1980. *Steringotrema robertpoulini* Pérez-Ponce de León, Anglade & Randhawa, 2018 falls within the *Steringophorus* Odhner, 1905 clade. In view of this the morphological and biological characteristics of species of *Steringophorus* and *Steringotrema* are discussed.

## Introduction

In 1999, we (Bray et al., 1999) published a phylogenetic tree exploring the possible deep-sea radiation of a group of fellodistomid digenean genera, mostly members of *Steringophorus* Odhner, 1905. All but one species was named. This unnamed species is described here from specimens from the same source as that sequenced in 1999. Several authors have used the named species in subsequent explorations of the fellodistomid phylogeny (Sun et al., 2014; Antar & Gargouri, 2016; Wee et al., 2017a; Wee et al., 2017b; Cutmore et al., 2018; Pérez-Ponce de León et al., 2018), but only one (Cribb et al., 2014) has included the unnamed species. In describing and naming this species, we hope that the sequences obtained from this rare and difficult to obtain deep-sea digenean will be used by all subsequent workers on fellodistomid phylogeny.

## Materials and methods

The host specimens were caught aboard the RRS *Challenger*, Cruise 43/1994, at the Goban Spur. The Goban Spur is an area of relatively shallower water that forms the southern margin of the Porcupine Seabight, a deep-water oceanic basin located on the continental margin to the west of southern Ireland. The fish were caught using an OTSB (semi-balloon otter trawl) with a single warp and dissected as soon as possible after being brought aboard. The live digeneans were washed in saline and fixed in Berland’s fluid for morphological study and 80% ethanol for molecular study. Whole-mounts were stained with Mayer’s paracarmine, cleared in beechwood creosote and mounted in Canada balsam. Measurements were made through a drawing tube on an Olympus BH-2 microscope, using a Digicad Plus digitising tablet and Carl Zeiss KS100 software adapted by Imaging Associates, and are quoted in micrometres. The following abbreviation is used: NHMUK, Natural History Museum, London, UK. The discussion of the hosts and distribution of *Steringophorus* and *Steringotrema* spp. is based on a database developed by Dr Thomas Cribb, at the University of Queensland, and maintained to date by RAB.

All 28S rDNA sequences used for this study were obtained from GenBank, although many were originally generated in our laboratory. Outgroup selection was informed by an unpublished 28S rDNA tree topology obtained for the preparation of Littlewood et al. (2015). Sequences were aligned using MAFFT version 7.157b (Katoh, 2008) with 1,000 cycles of iterative refinement and the genafpair algorithm. Ambiguously aligned positions were excluded by eye in Mesquite (Maddison & Maddison, 2018). MrModeltest v.2.3 (Nylander, 2004) was used to select a model of sequence evolution using the Akaike Information Criterion. Bayesian inference was performed using MrBayes version 3.2.2 (Ronquist et al., 2012). Two parallel runs were performed for 10 million generations and sampled every 1,000th generation. The burn-in was determined as the point at which the average standard deviation of split frequencies was < 0.01.

**Family Fellodistomidae Nicoll, 1909**

**Genus*Steringophorus*Odhner, 1905**

***Steringophorus merretti*n. sp.**

Syn. *Steringophorus* sp. ex *Cataetyx laticeps* of Bray et al. (1999)

*Type-host*: *Cataetyx laticeps* Koefoed (Ophidiiformes: Bythitidae).

*Type-locality*: Goban Spur (49°34'N, 13°11'W), Northeastern Atlantic; depth 1,654 m; 07.vii.1994).

*Type-specimens*: Holotype (NHMUK 1998.8.17.5); paratypes (NHMUK 1998.8.17.6-8.).

*Site in host*: Intestine.

*Representative DNA sequences*: GenBank: AJ405259 (*nad*1) and AJ405299 (28S rDNA).

*ZooBank registration*: To comply with the regulations set out in article 8.5 of the amended 2012 version of the *International Code of Zoological Nomenclature* (ICZN, 2012), details of the new species have been submitted to ZooBank. The Life Science Identifier (LSID) for *Steringophorus merretti* n. sp. is urn:lsid:zoobank.org:act:1678B67B-9482-4D49-9028-B48CCE3205E8.

*Etymology*: The species named for Dr Nigel Merrett in recognition of his major contributions to deep-sea biology and his expertise in abyssal trawling techniques.

### Description

**Fig. 1:**
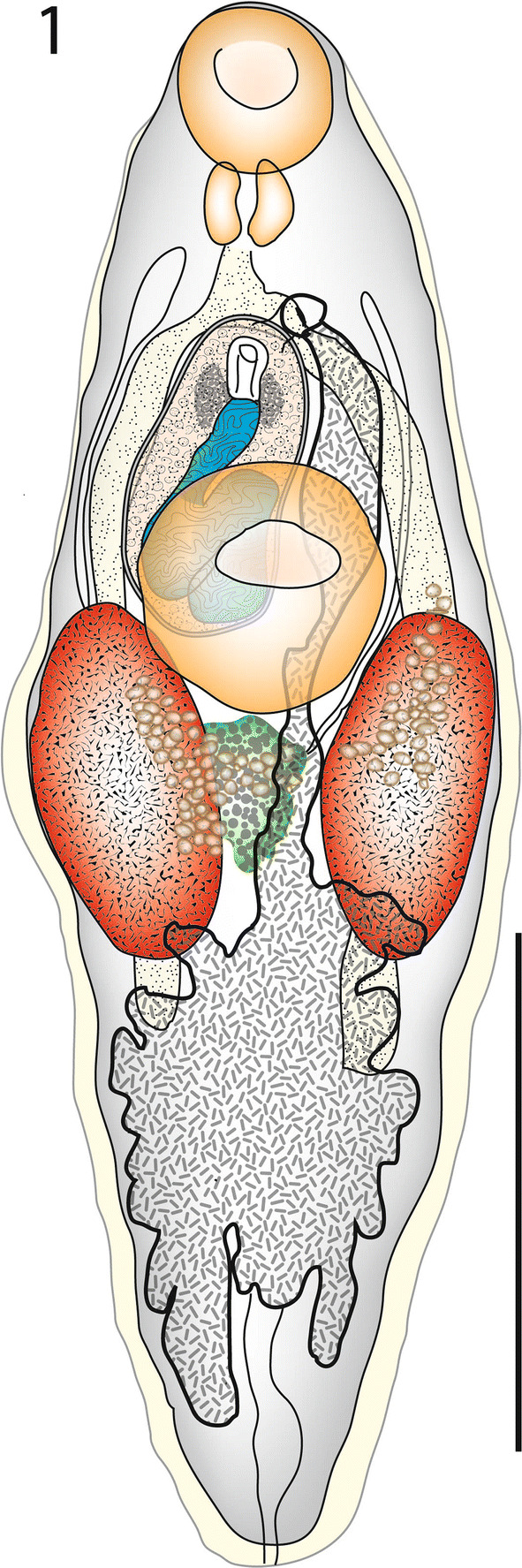
*Steringophorus merretti* n. sp. Ventral view of holotype. *Scale-bar*: 500 µm

**Fig. 2:**
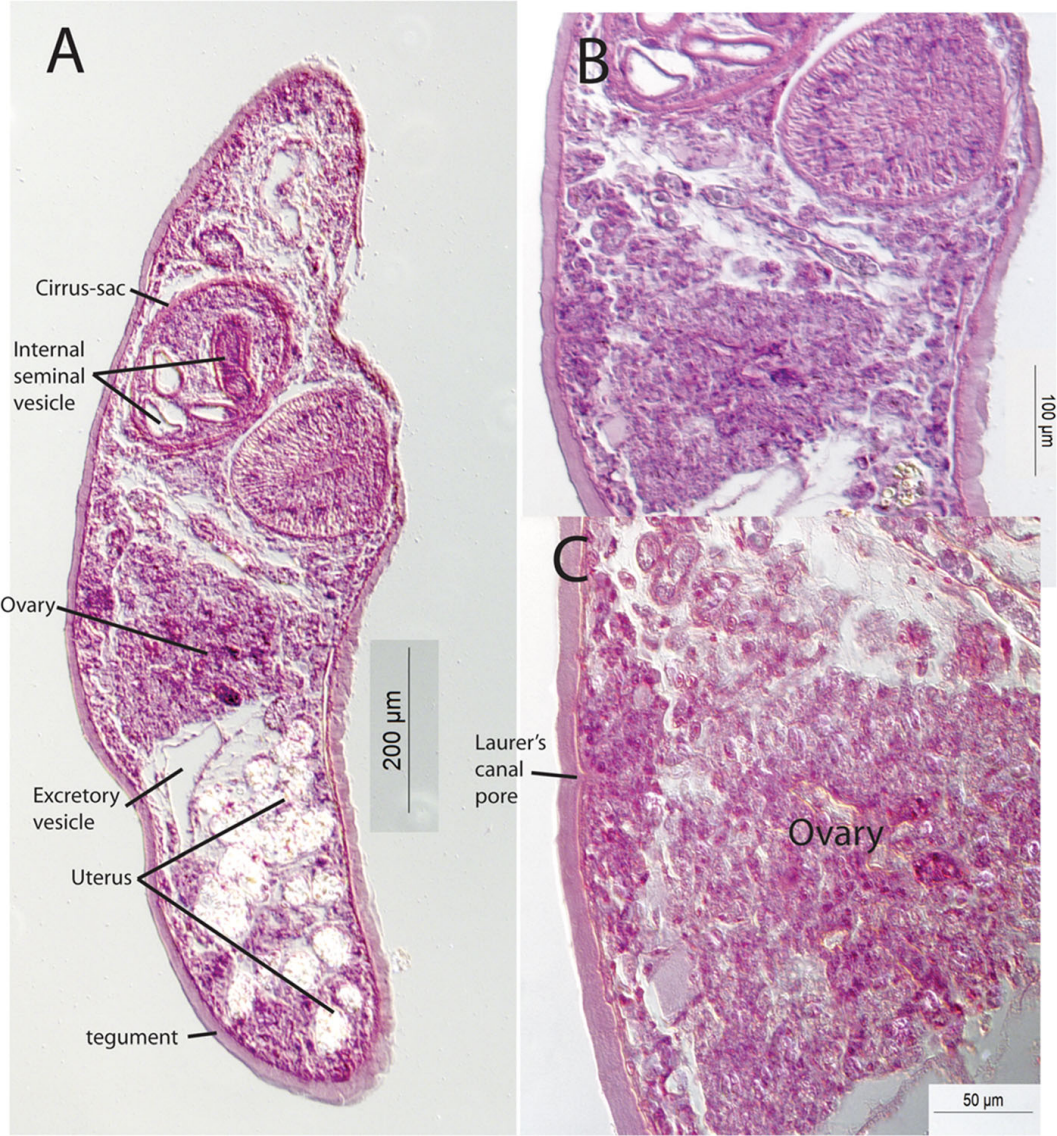
*Steringophorus merretti* n. sp. Sagittal sections. A, Complete worm; B, Ventral sucker and anterior hindbody; C, Ovary and Laurer’s canal opening

[Based on 6 whole-mounted, measured specimens and 2 sagittally sectioned specimens; Figs. 1, 2]. Body fusiform, rounded at each end; 1,260–1,664 × 420–525 (1,445 × 472); width 28.9–37.7 (32.8)% of length (Fig. 1). Tegument unarmed, but particularly thick, especially posteriorly (Fig. 2a). Oral sucker (OS) rounded, subterminal, 155–172 × 148–182 (161 × 168); length (L) 10.3–12.4 (11.2)% of body-length (BL). Ventral sucker (VS) 218–271 × 209–278 (252 × 252); length 16.3–18.4 (17.5)% of BL. Sucker ratios: length 1:1.40–1.68 (1.57); width (SWR) 1.36–1.69 (1.50). Forebody length (FBL) 352–471 (418), 25.5–32.7 (29.0)% of BL. Prepharynx not detected. Pharynx (Ph) oval, 69–100 × 83–91 (85 × 87); length 5.02–7.33 (5.92)% of BL. OS to Ph width ratio 1: 0.49–0.61 (0.52). Oesophagus (Oes) 31–97 (62) long, 2.44–7.02 (4.26)% of BL. Intestinal bifurcation (IB) in mid-body, 52–160 (129) from ventral sucker, 14.8–36.6 (30.3)% of FBL. Caeca blind, fairly short, terminate in anterior post-testicular region 345–523 (453) from posterior extremity; post-caecal distance (PCD) 27.4–32.5 (30.9)% of BL.

Cirrus-sac (CS) broad, oblong, thick-walled, 273–341 × 126–78 (312 × 153); length 20.5–23.4 (21.6)% of BL. Internal seminal vesicle broad, tubular, convoluted, in posterior two-thirds of cirrus-sac. Prostatic cells accumulate around junction of seminal vesicle and ejaculatory duct. Ejaculatory duct broad, pocketed, may be protruded through genital pore as cirrus. Genital atrium wide, fairly shallow. Genital pore median or slightly sinistral, bifurcal. Testes (TT) elongate-oval, smooth, symmetrical, in anterior hindbody and overlapping ventral sucker, close to lateral margins, 232–361 × 109–202 (303 × 156); length 17.3–24.6 (21.1)% of BL. Post-testicular region (PTR) 473–693 (575) long, 37.5–41.9 (39.7)% of BL.

Ovary (Ov) follicular, overlaps ventral sucker and testes, not clearly delineated in whole-mounts, histologically rather amorphous (Fig. 2), 183–229 × 133–178 (202–156); length 12.6–15.5 (13.9)% of BL. Seminal receptacle uterine. Laurer’s canal opens dorsal to ovary (Fig. 2c). Uterus reaches to 119–205 (176) from posterior extremity; post-uterine distance (PUD) 8.30–15.8 (12.3)% of BL. Metraterm of similar length to cirrus-sac, wide, thick-walled. Eggs tiny, 13–18 × 7–10 (15 × 9), possibly deformed but same in all specimens. Vitellarium in form of small lateral fields of small follicles, overlaps posterior margin of ventral sucker but does not reach posterior to testes; fields (VFL) 158–216 (183) long, 10.6–15.0 (12.7)% of BL. Pre-vitelline distance (PrVD) 460–577 (533), 33.9–40.1 (37.0)% of body-length; post-vitelline distance (PoVD) 592–919 (733), 46.3–55.2 (50.5)% of BL.

Excretory pore terminal. Vesicle Y-shaped; branching point obscured by eggs; arms reach just pre-bifurcal.

Differential diagnosis

This is the only *Steringophorus* species reported from a bythitid and is notable for its tiny eggs, much smaller than reported for any other species of the genus. *Steringophorus magnus* Manter, 1934, with eggs 30–32 × 14–17 (Manter, 1934), and *Steringophorus haedrichi* Bray & Campbell, 1995, with eggs 32–41 × 16–22 (Bray & Campbell, 1995; Bray & Gibson, 1998), are those species with the next smallest eggs. It may be that they are deformed, or poorly formed, in the material of the new species, as there is a lot of un-attached shell material in the uterus, but all specimens have similar-sized eggs. *Steringophorus merretti* n. sp. has a relatively larger cirrus-sac (at length 20.5–23.4% of BL) than any other species for which cirrus-sac measurements are available.

*Steringophorus merretti* n. sp. also differs from the 14 other congeners thus:

*Steringophorus arntzi* Zdzitowiecki, 1997, from notothenioid fishes in the Weddell Sea, lacks an Oes, TT are smaller (*c.*14% of BL), has a shorter PCD (*c.*15% of BL), a shorter PTR (*c.*20–34% of BL), a shorter PoVD (*c.*31–39% of BL) and a shorter PUD (*c.*3% of BL) (Zdzitowiecki, 1997; Zdzitowiecki, 2002a, 2002b).

*Steringophorus blackeri* Bray, 1973, from the alepocephalid *Xenodermichthys copei* (Gill) from the North Atlantic, is genetically distinct (see below), distinctly larger at 2,200–9,000 long, has a shorter FBL (*c.*16–22% of BL), smaller VSL (*c.*5–9% of BL), smaller SWR (*c.*1:0.96–1.16), smaller TT (at 2–7% of BL), longer PTR (*c.*55–70% of BL), a smaller Ov (at 3–8% of BL), shorter PrVD (*c.*15–25% of BL) and a longer vitellarium (*c.*19–27% of BL) (Bray, 1973; Markle & Wenner, 1979; Bray & Gibson, 1980; Campbell, 1983; Aleshkina & Gaevskaya, 1985; Bray & Campbell, 1995; Bray et al., 1999).

*Steringophorus congeri* Shen, 1987 from congrid, bathylagid and microstomatid fishes in the north-western Pacific Ocean, is distinctly larger at 4,040–10,268 long, a longer FBL (*c.*35% of BL), smaller suckers (OSL *c.*8–9%, VSL *c.*10–11% of BL), smaller Ph (length *c.*3–4% of BL), longer Oes (*c.*8–10% of BL), a shorter PCD (*c.*15% of BL), smaller TT (length 7–8% of BL), smaller Ov (*c.*7–8 of BL), a longer vitellarium (*c.*30% of BL) and a shorter PoVD (*c.*40% of BL) (Shen, 1987; Kuramochi, 2009).

*Steringophorus dorsolineatus* (Reimer, 1985) Bray, 1995 (syn. *Occultacetabulum dorsolineatum* Reimer, 1985) from ipnopidae fishes of the genus *Bathypterois* in the south-western Indian Ocean, the Atlantic and probably the Mediterranean Sea, is genetically distinct (see below), has a smaller VSL (length *c.*8–14% of BL), smaller SWR (1:0.84–1.06), the Oes is generally longer (5–10% of BL), a shorter PCD (*c.*13–14% of BL), smaller TT (length 7–9% of BL), smaller Ov (*c.*6–8 of BL), a longer vitellarium (*c.*21–26% of BL) and a shorter PoVD (*c.*34–36% of BL) (Reimer, 1985; Bray, 1995; Bray & Kuchta, 2006; Mateu et al., 2014).

*Steringophorus foliatus* (Yamaguti, 1970) Bray & Gibson, 1980 (syn. *Callodistomoides foliatus* Yamaguti, 1970) from an unidentified inermiid fish of the genus *Emmelichthyops* off Hawaii, has a smaller OSL (*c.*7% of BL), smaller PhL (*c.*3.5% of BL), a shorter PCD (*c.*10% of BL) and smaller TT (length 8–11% of BL) (Yamaguti, 1970). Randall (2007) does not include any *Emmelichthyops* species in his book on Hawaiian marine fishes. On the other hand, he does include three species of *Emmelichthys*. These species are found at bathyal depths, with the golden rover *E. scintillans* (Jordan & Thompson) reported to 606 m, so it could possibly harbour *Steringophorus*.

**Fig. 3:**
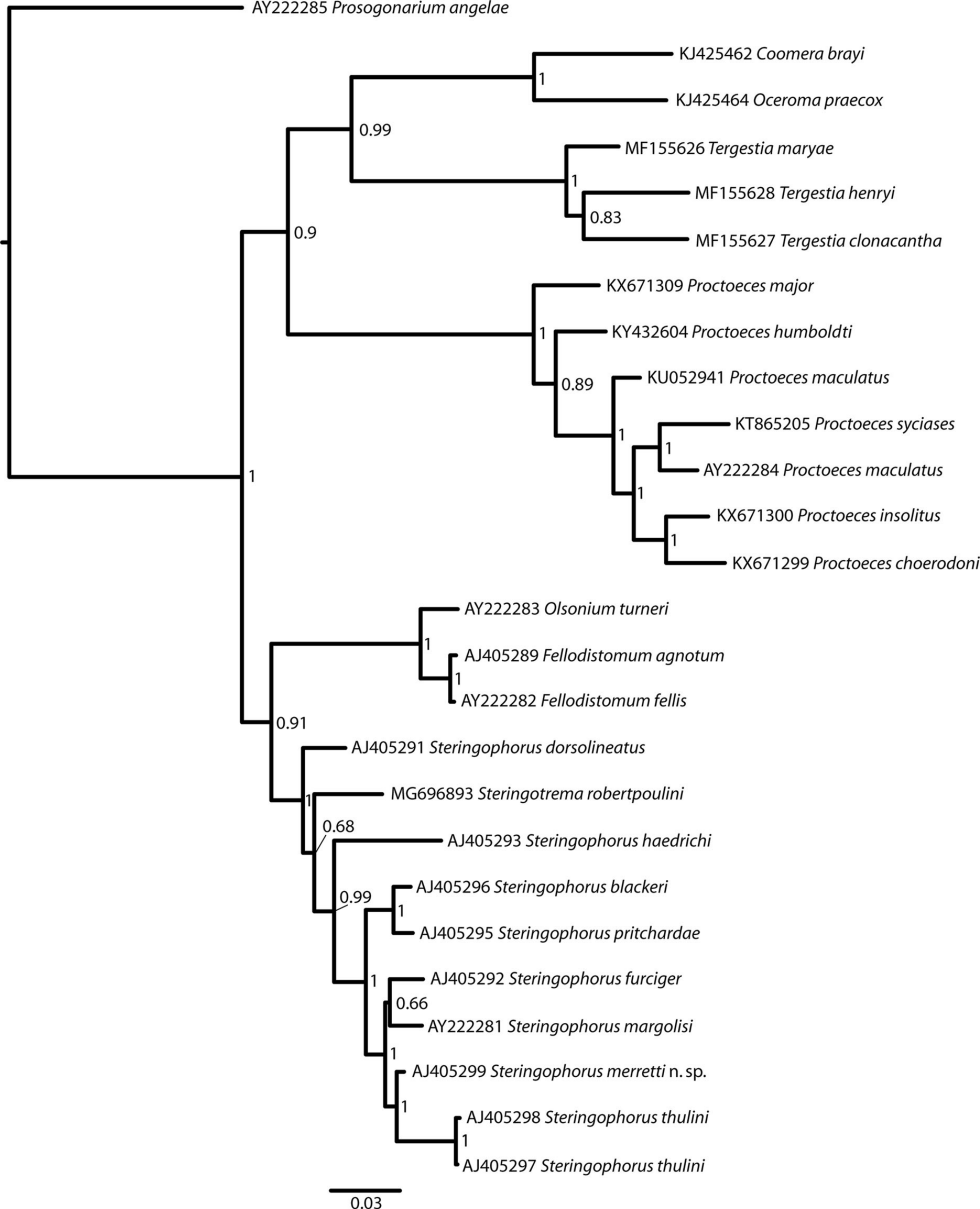
Bayesian analysis of available fellodistomid 28S rDNA sequences. Tree constructed using MrBayes version 3.2.1 under the GTR+I+G model of nucleotide evolution; 10,000,000 generations, 5,000,000 generations ‘burn-in’

*Steringophorus furciger* (Olsson, 1868) Odhner, 1905 (syns *Distoma furcigerum* Olsson, 1868; *Leioderma furcigerum* (Olsson, 1868) Stafford, 1904; *Fellodistomum furcigerum* (Olsson, 1868) Yamaguti, 1954) from many fish species, particularly pleuronectids, from the northern parts of the Atlantic and Pacific Oceans, is genetically distinct (Fig. 3). It is a commonly recorded species and has been described many times (Olsson, 1868; Levinsen, 1881; Odhner, 1905; Lebour, 1908; Yamaguti, 1934; Linton, 1940; Miller, 1941; Polyanski, 1955; Strelkov, 1960; Bray & Gibson, 1980; Machida, 1988; Bray & Campbell, 1995). It is debatable if all the records attributed to this species are accurate, but apart from the egg and cirrus-sac size difference, the testes tend to be smaller (*c.*7–15% of BL) and the vitelline length is larger (*c.*19–26% of BL). Bray & Gibson (1980) and Machida (1988) discussed the variation found in this species, and particularly that of the egg-size, but all published measurements are distinctly larger to much larger than in *S. merretti* n. sp.

*Steringophorus haedrichi* Bray & Campbell, 1995 from ophidiid fishes in the north-eastern Atlantic, is genetically distinct (see below), is distinctly larger at 3,514–5,692, with a smaller VSL (*c.*11–14% of BL), a shorter PCD (*c.*23–24% of BL), smaller TT (length 10–13% of BL) and a smaller Ov (*c.*8–10 of BL) (Bray & Campbell, 1995; Bray & Gibson, 1998; Bray et al., 1999).

*Steringophorus liparidis* Zdzitowiecki, 1997 from liparid and muraenolepidid fishes from the Weddell and Ross Seas, grows distinctly larger at 2,000–6,418 long, has a smaller VSL (*c.*13–14% of BL), a shorter PCD (*c.*7–11% of BL), shorter PTR (*c.*21–35% of BL) and a longer vitellarium (*c.*23–24% of BL) (Zdzitowiecki, 1997; Sokolov & Gordeev, 2015).

*Steringophorus magnus* Manter, 1934 from “unidentified eel-like fish” off Florida, is distinctly larger at 6,080–7,220 long, with a shorter FBL (*c.*20% of BL), smaller OSL (*c.*6–7% of BL), smaller VSL (*c.*13–15% of BL), larger SWR (1:2.18–2.45), longer PCD (*c.*38% of BL) and longer PTR (*c.*51% of BL) (Manter, 1934).

*Steringophorus margolisi* Bray, 1995 from the ophidiid fish *Spectrunculus grandis* (Günther), in the north-eastern Atlantic, is genetically distinct (see below), is distinctly larger at 4,659–5,840 long, narrower (width 22–23% of BL), shorter FBL (9–23 of BL), smaller OSL (*c.*7–9% of BL), smaller VSL (*c.*8–9% of BL), smaller SWR (1:1.23–1.33), shorter PCD (8% of BL), smaller TT (length 7% of BL), longer PTR (*c.*56–61% of BL), smaller OvL (*c.*5% of BL), smaller PrVD (*c.*27% of BL) and longer VFL (*c.*27% of BL) (Bray, 1995; Bray et al., 1999; Olson et al., 2003; Bray & Kuchta, 2006).

*Steringophorus melanostigmum* (Noble & Orias, 1975) (syns. *Fellodistomum melanostigmum* Noble & Orias, 1975; *Benthotrema melanostigmi* Parukhin & Lyadov, 1979) from zoarcid fishes of the genus *Melanostigma* in the Pacific, Indian and Atlantic Ocean, grows distinctly larger at 1,435–4,920 long, with a smaller VSL (*c.*7–9% BL), smaller SWR (1:0.97–1.12), shorter Oes (*c.*0–2% of BL), testes usually smaller (*c.*4–18% of BL), longer PTR (*c.*48–50% of BL), usually smaller OvL (*c.*4–16% of BL), shorter PrVD (*c.*15–28% of BL) and a longer VFL (*c.*24–31% of BL). *Benthotrema melanostigmi* is presently considered a synonym of *S. melanostigmum*, but certain characters such as the size of the gonads and the anterior extent of the vitellarium suggest that it may be distinct (Noble & Orias, 1975; Markle & Wenner, 1979; Parukhin & Lyadov, 1979).

*Steringophorus pritchardae* (Campbell, 1975) Bray & Gibson, 1980 (syn. *Abyssotrema pritchardae* Campbell, 1975) mainly from alepocephalid fishes of the genus *Alepocephalus*, with one report from a morid, from the north Atlantic Ocean, is genetically distinct (see below), is distinctly larger at 3,900–13,800 long, with a shorter FBL (*c.*20–22% of BL), smaller OSL (*c.*5–10% of BL), smaller VSL (*c.*4–8% of BL), smaller SWR (*c.*1:0.81–1.06), smaller PCD (*c.*22% of BL), smaller TTL (*c.*4–9% of BL), smaller OvL (*c.*4–6% of BL), smaller PrVD (25–28% of BL) and a longer VFL (*c.* 26–35% of BL) (Campbell, 1975; Bray & Gibson, 1980; Campbell et al., 1980; Gaevskaya & Aleshkina, 1983; Bray et al., 1999; Bray & Kuchta, 2006; Gordeev et al., 2019).

*Steringophorus profundus* Manter, 1934 from the argentinid fish *Argentina striata* Goode & Bean off Florida, is slightly wider (*c.*40–45% of BL), with a shorter PCD (*c.*40% of BL), longer VFL (*c.*23% of BL) and shorter PoVD (*c.*42% of BL) (Manter, 1934).

*Steringophorus thulini* Bray & Gibson, 1980 (syn. *Callodistomoides foliatus* of Zubchenko, 1975) from macrourids (mainly) and lotids in the northern Atlantic Ocean, is genetically distinct (see below), has longer caeca (PCD 16–19% of BL), smaller TT (12–16% of BL), shorter PTR (26–36% of BL), longer VFL (22–25% of BL) and shorter PoVD (36–45% of BL) (Zubchenko, 1975; Bray & Gibson, 1980; Bray, 1995; Bray et al., 1999; Kellermanns et al., 2009).

**Molecular phylogeny**

Molecular results indicate that *Steringophorus merretti* is distinct from all other *Steringophorus* species for which sequences are available. A molecular phylogeny, inferred from 28S rDNA sequences obtained from GenBank (Table 1), is presented in Fig. 3. This is similar to that published by Pérez-Ponce de León et al. (2018) but includes more shallow waters species of the genera *Tergestia* Stossich, 1899 and *Proctoeces* Odhner, 1911 in addition to *S. merretti*. In Table 1, we have included bathymetric data for these species, when available, which, in conjunction with the tree, shows that the family Fellodistomidae consists of two readily distinguished clades, characterised by the bathymetry of the species. The main caveat with this finding is the position of the shallow-water form *Steringotrema robertpoulini* Pérez-Ponce de León, Anglade & Randhawa, 2018. This species is from the New Zealand sole *Peltorhamphus novaezeelandiae* Günther (Pleuronectidae) off South Island, New Zealand (Pérez-Ponce de León et al., 2018). According to Froese & Pauly (2019) it ‘inhabits shallow waters, generally at depth of less than 50 m’. It is the only member of its genus for which molecular data are available, so it is not currently possible to state that this a genuinely anomalous position or that a group of related species have invaded shallow water.

**Table 1:** Data on the 28S rDNA sequences used in the phylogenetic analysis

GenBank ID	Parasite	Host	Locality	Depth	Reference
AY222285	*Prosogonarium angelae* Cribb & Bray, 1994	*Euristhus lepturus*	Moreton Bay, Queensland, Australia	Shallow	Olson et al. (2003)
KJ425462	*Coomera brayi* Dove & Cribb, 1995	*Monodactylus argenteus*	Moreton Bay, Queensland, Australia	Shallow	Cribb et al. (2014)
KJ425464	*Ocerama praecox* (Walker, 1971)	*Scorpis lineolata*	Moreton Bay, Queensland, Australia	Shallow	Cribb et al. (2014)
MF155626	*Tergestia maryae* Wee, Cutmore, Yong & Cribb, 2017	*Alepes apercna*	Moreton Bay, Queensland, Australia	Shallow	Wee et al. (2017b)
MF155628	*Tergestia henryi* Wee, Cutmore, Yong & Cribb, 2017	*Pantolabus radiatus*	Moreton Bay, Queensland, Australia	Shallow	Wee et al. (2017b)
MF155627	*Tergestia clonacantha* Manter, 1963	*Hyporhamphus regularis ardelio*	Moreton Bay, Queensland, Australia	Shallow	Wee et al. (2017b)
KX671309	*Proctoeces major* Yamaguti, 1934	*Monodactylus argenteus*	Moreton Bay, Queensland	Shallow	Wee et al. (2017a)
KY432604	*Proctoeces humboldti* George-Nascimento & Quiroga, 1983	*Sicyases sanguineus*	Coquimbo, Chile	Shallow	Oliva et al. (2018)
KU052941	*Proctoeces maculatus* (Looss, 1901)	*Sabella pavonina*	Bizerte Lagoon, Tunisia	Shallow	Antar & Gargouri (2016)
KT865205	*Proctoeces syciases* Oliva, Valdivia, Cárdena, Muñoz, Escribano & George-Nascimento, 2018	*Sicyases sanguineus*	Iquique, Chile	Shallow	Oliva et al. (2018)
AY222284	*Proctoeces maculatus*	*Archosargus probatocephalus*	Gulf of Mexico, Mississippi, USA	Shallow	Olson et al. (2003)
KX671300	*Proctoeces insolitus* (Nicoll, 1915)	*Acanthopagrus australis*	Moreton Bay, Queensland, Australia	Shallow	Wee et al. (2017a)
KX671299	*Proctoeces choerodoni* Wee, Cribb, Bray & Cutmore, 2017	*Choerodon cyanodus*	Off Heron Island, Queensland, Australia	Shallow	Wee et al. (2017a)
AY222283	*Olsonium turneri* Bray & Gibson, 1980	*Alepocephalus agassizii*	Porcupine Seabight, NE Atlantic	2,441 m	Olson et al. (2003)
AJ405289	*Fellodistomum agnotum* Nicoll, 1909	*Anarhichas lupus*	North Sea	135 m	Bray et al. (1999)
AY222282	*Fellodistomum fellis* (Olsson, 1868)	*Anarhichas lupus*	North Sea	135 m	Olson et al. (2003)
AJ405291	*Steringophorus dorsolineatus* (Reimer, 1985)	*Bathypterois dubius*	Goban Spur, NE Atlantic	1,541m	Bray et al. (1999)
MG696893	*Steringotrema robertpoulini* Pérez-Ponce de León, Anglade & Randhawa, 2018	*Peltorhamphus novaezeelandiae*	South Island, New Zealand	Shallow	Pérez-Ponce de León et al. (2018)
AJ405293	*Steringophorus haedrichi* Bray & Campbell, 1995	*Spectrunculus grandis*	Goban Spur, NE Atlantic	2,570 m	Bray et al. (1999)
AJ405296	*Steringophorus blackeri* Bray, 1973	*Xenodermichthys copei*	Goban Spur, NE Atlantic	960 m	Bray et al. (1999)
AJ405295	*Steringophorus pritchardae* (Campbell, 1975)	*Alepocephalus rostratus*	Goban Spur, NE Atlantic	1,625 m	Bray et al. (1999)
AJ405292	*Steringophorus furciger* (Olsson, 1868)	*Limanda limanda*	Off St Abbs, North Sea	59 m	Bray et al. (1999)
AY222281	*Steringophorus margolisi* Bray, 1995	*Spectrunculus grandis*	Rockall Trough, NE Atlantic	1,745–1,760 m	Olson et al. (2003)
AJ405299	*Steringophorus merretti* n. sp.	*Cataetyx laticeps*	Goban Spur, NE Atlantic	1,654 m	Bray et al. (1999)
AJ405298	*Steringophorus thulini* Bray & Gibson, 1980	*Coryphaenoides leptolepis*	Porcupine Abyssal Plain	4,100 m	Bray et al. (1999)
AJ405297	*Steringophorus thulini*	*Coryphaenoides mediterranea*	Rockall Trough, NE Atlantic	1,745–1,760 m	Bray et al. (1999)

Pleuronectiforms are not rare hosts of *Steringophorus* spp. (see below), but the vast majority of reports are of the species *S. furciger*, a species which is thought to range into deeper waters (Bray, 2004). *Steringophorus merretti* is resolved as the sister species of another deep-sea species, *S. thulini*, and is embedded within a clade of deep-sea species.

***Steringophorus*Odhner, 1905**

**Fig. 4:**
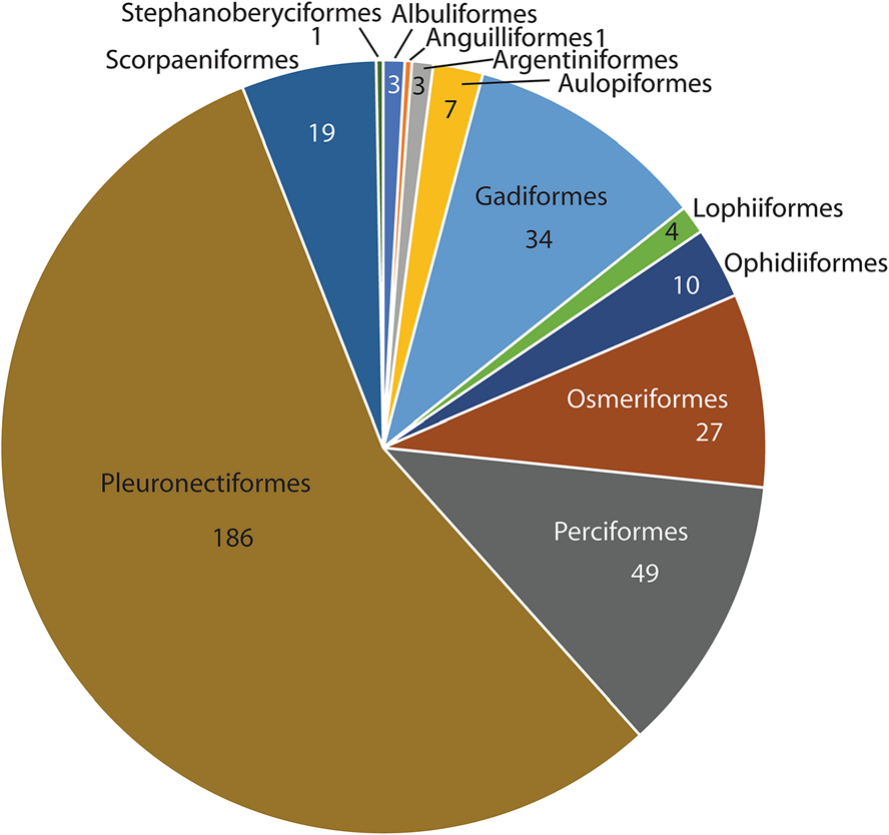
Pie graph showing records from host orders: All *Steringophorus* records

**Fig. 5:**
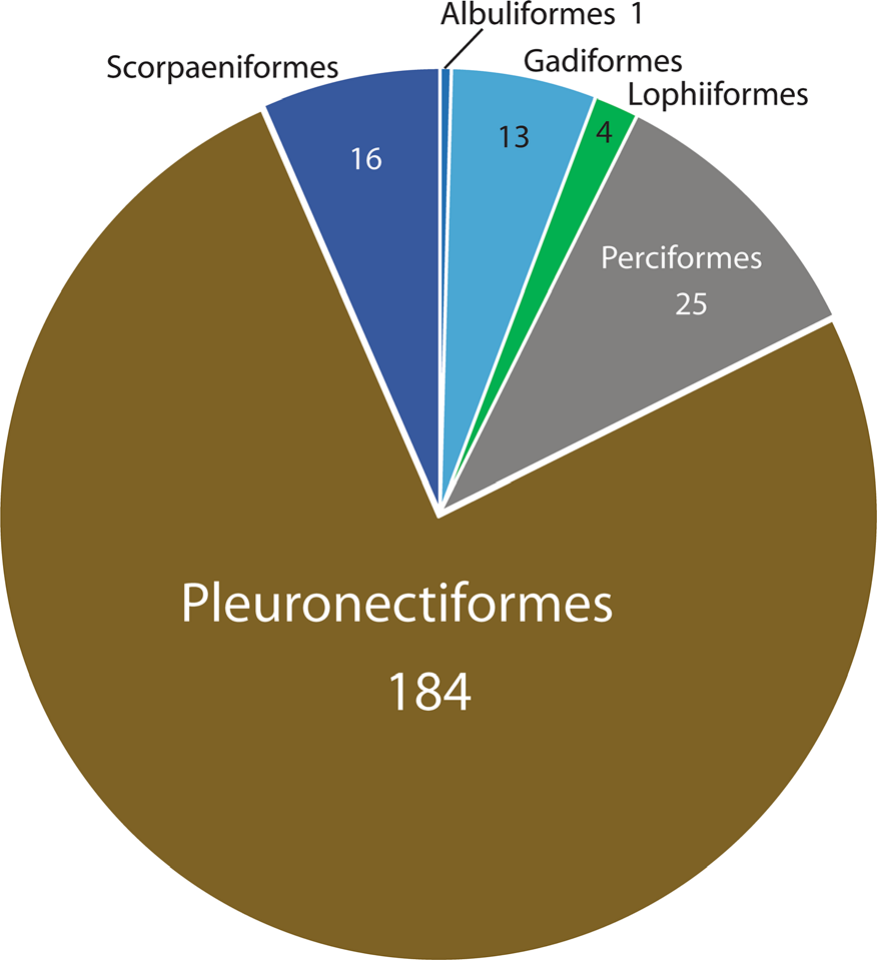
Pie graph showing records from host orders: All *S. furciger* records

**Fig. 6:**
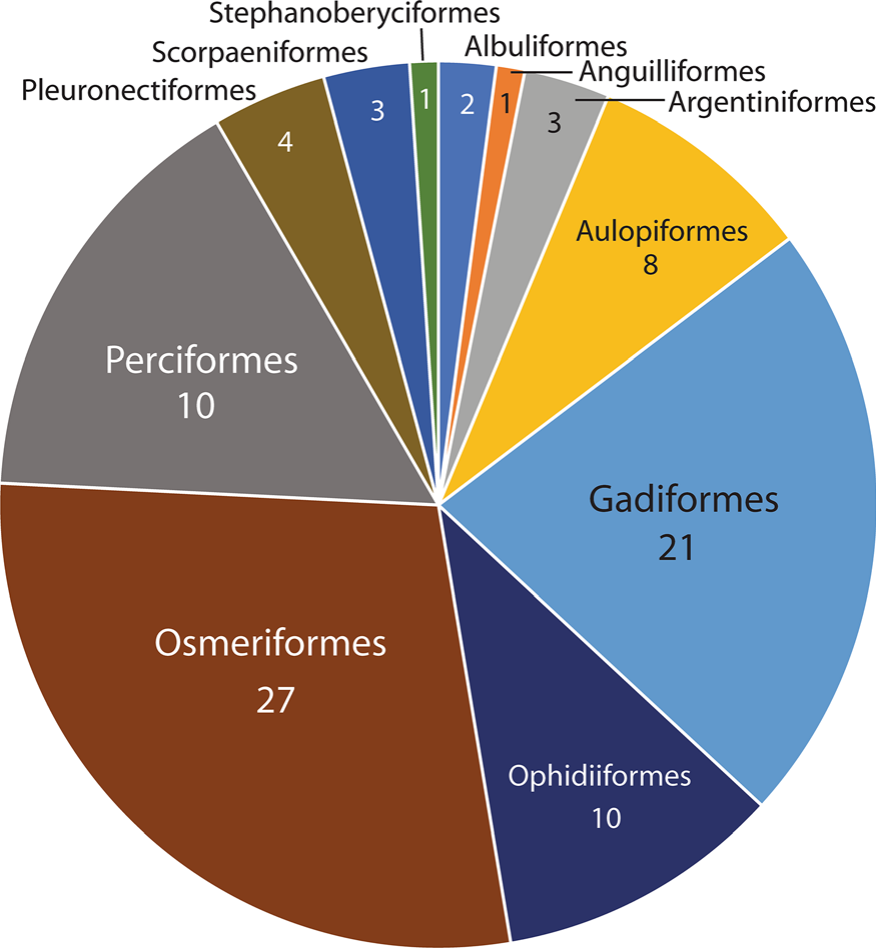
Pie graph showing records from host orders: All non-*furciger* records

We are aware of 338 host/parasite reports of the 15 members of this genus, 242 (72%) of which are of *S. furciger*. Therefore, in any discussion of the biological characteristics of the genus, the predominance of data on this species must be considered. If we look at the fish orders harbouring species of *Steringophorus* (Fig. 4), we see a predominance of reports in the pleuronectiforms (55%). Considering *S. furciger* (Fig. 5) only, we see that 76% of records are from this order, but non-*furciger* species have about 4% of records from this order, and none are identified to species and may well be *S. furciger* (see Arthur & Albert, 1994; Chambers, 2008). The predominant host orders for non-*furciger* species are the deep-water taxa, the Osmeriformes (with 28% of records) and the Gadiformes (22%) (Fig. 6). Other predominantly deep-sea orders constitute the majority of the other reports: Ophidiiformes (11%), Aulopiformes (8%), Scorpaeniformes (3%) and Argentiformes (3%). Even the perciform hosts reported for *Steringophorus* spp. (10% for *S. furciger*, 15% for non-*furciger* species) are almost entirely zoarcids (with many upper bathyal species) or Antarctic notothenioids (Artedidraconidae, Bathydraconidae). The genus is, therefore, predominantly found in deep-sea or demersal cold-water fishes.

**Fig. 7:**
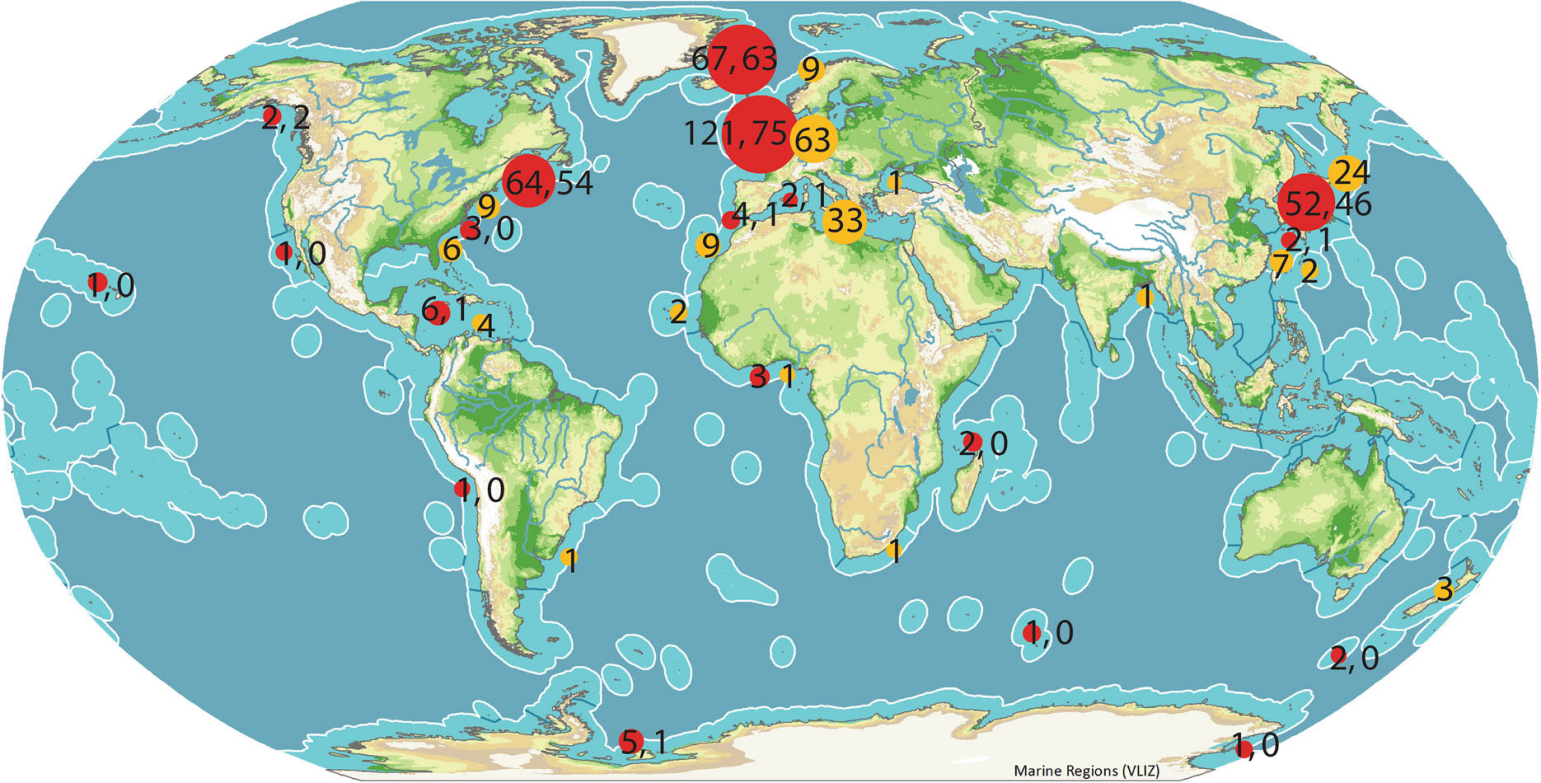
Global distribution of *Steringophorus* and *Steringotrema* records plotted on Spalding et al. (2007) ecoregions. Red circles, *Steringophorus*; orange circles *Steringotrema*

The distribution patterns are mapped in Fig. 7, showing the ecoregions of Spalding et al. (2007), with the red circles referring to *Steringophorus* spp. and the orange circles to *Steringotrema* spp. The numbers in the red circles (which are not to strictly to scale but give some impression of the number of records) are the number of records of all *Steringophorus* species, followed by the number of *S. furciger* records separated by a comma. All reports of *S. furciger*, and the vast majority of all records, are in the high northern latitudes. The infrequent reports from elsewhere may be affected by sampling effort but are also likely to be indicative of a genuine dearth, as findings of *Steringophorus* spp. are virtually absent from among the many reports of digeneans from tropical and subtropical regions. Existing reports from apparently warm water regions should be examined closely. For example, the report of specimens from ecoregion 20 (Western Indian Ocean) is of *S. dorsolineatus* from the deep-sea ipnopid *Bathypterois phenax* Parr (Reimer, 1985). The three records in ecoregion 17 (Gulf of Guinea) are of parasites of members of the deep-sea family Alepocephalidae (Gaevskaya & Aleshkina, 1983; Aleshkina & Gaevskaya, 1985). The report from off northern Chile (ecoregion 45, Warm Temperate Southeastern Pacific) is of an unnamed species from the macrourid *Coryphaenoides ariommus* Gilbert & Thompson (Nacari & Oliva, 2016). All reports with identified hosts from the Gulf of Mexico (ecoregion 6, Warm Temperate Northwest Atlantic, and ecoregion 12, Tropical Northwestern Atlantic) are from the deep-sea families Ophidiidae, Argentinidae or Alepocephalidae (Manter, 1934, 1947; Harris & Dronen, 1999). There is no evidence that species of *Steringophorus* occur in shallow-water or reef fishes in lower latitudes.

***Steringotrema*Odhner, 1911**

As *Steringotrema robertpoulini* is, apparently, embedded within *Steringophorus*, according to our tree (Fig. 3) and that of Pérez-Ponce de León et al. (2018), it seems appropriate to discuss this genus and its relationship with *Steringophorus*. According to Bray (2002), the genera are distinguished by the vitelline configuration of their constituent species, the shape of the ovary and the excretory vesicle. In *Steringotrema* spp. the vitellarium is mainly, or has a significant proportion, in the forebody, whereas in *Steringophorus* the vitellarium is mainly in the hindbody. The ovary is multilobate in *Steringophorus* spp., but in *Steringotrema* spp. the ovary is entire or tri-lobed. The excretory vesicle is Y-shaped in *Steringophorus* spp. and V-shaped in *Steringotrema* spp. Individual species in these genera exhibit some variation in these features. For example, in *Steringophorus dorsolineatum,* the ovary was originally described as smooth (“glattrandig”) (Reimer, 1985) and later as “weakly multilobate” (Bray, 1995). It remains to be seen how useful these characters are in reflecting the phylogenetic relationships within this family. The relationships of these genera and the closely similar genera *Lomasoma* Manter, 1935, *Megenteron* Manter, 1934, *Prudhoeus* Bray & Gibson, 1980 and *Steringovermis* Bray, 2004 await molecular study.

**Fig. 8:**
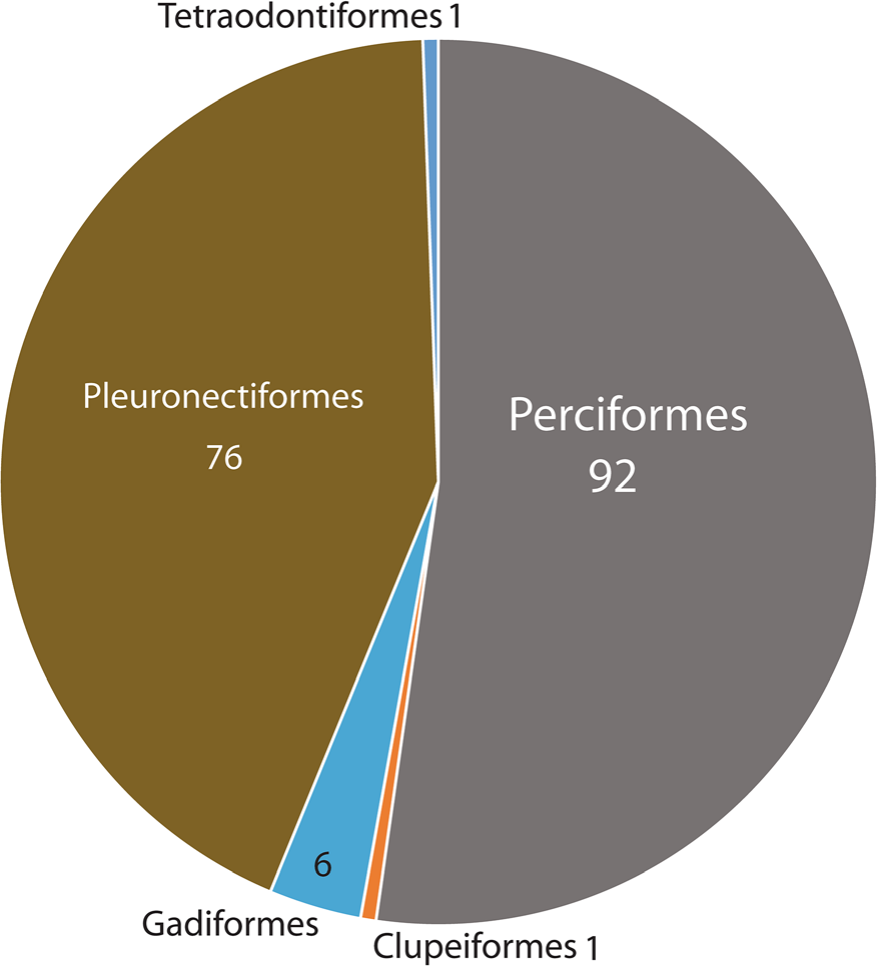
Pie graph showing records of *Steringotrema* spp. from different host orders

We are aware of 176 host/parasite records of 12 *Steringotrema* spp. There is no single predominant species, but three species make up 81% of records. These are the northern temperate species *S. pagelli* (van Beneden, 1871) (35%), *S. ovacutum* (Lebour, 1908) (28%) and *S. divergens* (Rudolphi, 1809) (18%). Perciformes (53%) and Pleuronectiformes (43%) are the predominant host groups (Fig. 8). There are six records from Gadiformes, four of which are not identified to species, and there are single records of named species from Clupeiformes and Tetraodontiformes. In contrast to *Steringophorus*, the majority of reports from perciform hosts are from the shallow water groups Sparidae (50%) and Blennidae (23%). Kellermanns et al. (2009) found an unnamed *Steringotrema* in the macrourid *Coryphaenoides mediterraneus* (Giglioli) from the Charlie-Gibbs Fracture Zone on the Mid-Atlantic Ridge, with a range of depths for the host species (but not necessarily for the parasite) of 1,700–3,050 m. Nevertheless, the finding in a macrourid is certainly worthy of note as an anomalous finding for the parasite. Other records of *Steringotrema* spp. in macrourids are by Palm & Klimpel (2008) (‘*Steringotrema pagelli*ʼ in *Macrourus berglax* Lacépède, depth 278–423 m, Irminger Sea) and Constenla et al. (2015) (*Steringotrema* sp., in *Trachyrincus scabrus* (Rafinesque), depth 574–1,000 m, Western Mediterranean). Further deep-water records are by Kuramochi (2014) (*Steringotrema* sp. in *Bothrocara hollandi* (Jordan & Hubbs), Zoarcidae, depth 1,394–1,400 m, Sea of Japan) and Dallarés et al. (2016) (*Steringotrema* sp. in *Phycis blennoides* (Brünnich), Phycidae, 400–1,000 m, Balearic Sea). In no cases were descriptions given. Nevertheless, most evidence points to *Steringotrema* species being denizens of shallow-waters, whereas *Steringophorus* species are adapted to deeper-waters.

*Steringotrema* spp. are distributed mainly in cold to temperate waters, with most records in the northern parts of the Atlantic and Pacific Oceans (Fig. 7). They may be more tolerant of warmer waters than *Steringophorus* spp.; for example, it is much more commonly reported in the Mediterranean Sea (i.e. ecoregion 4). The few reports from warm waters are, however, not definitive. Fischthal & Thomas (1968) reported, but did not describe, three immature specimens of *Markevitschiella* sp. (a genus widely considered a synonym of *Steringotrema*) from *Pagrus caeruleostictus* (Valenciennes) (as *P. ehrenbergi* Valenciennes) off Ghana (ecoregion 17, Gulf of Guinea). Not enough information is available to be confident that this was a correct identification. *Steringotrema divergens* (Rudolphi, 1809) has been reported in *Pagellus bogaraveo* (Brünnich) off Senegal (ecoregion 16, West African Transition) (Fischthal & Thomas, 1972; Vassiliadès, 1982). These are the only records of this species from sparids but there are no descriptions.

## Conclusion

It is more satisfactory for phylogenies to include named and described species and, to this end, we have described here a species for which some molecular data are available. This species is not morphologically highly distinct, but it is from a rarely seen and difficult to obtain host. The only other report of a digenean from this species is that of the hemiurid *Merlucciotrema praeclarum* (Manter, 1934) by Bray (1996) from the same individual fish. This is a glimpse into the poorly known digenean fauna of the deep-sea, which is worthy of much more sustained study.
